# Multifaceted assessment of modifying and rejuvenating asphalt binders with recycled waste plastic and engine oil

**DOI:** 10.1038/s41598-025-25951-z

**Published:** 2025-12-02

**Authors:** Aya A. El-Sherbeni, Alaa R. Gabr, Sherif M. El-Badawy, Ahmed M. Awed

**Affiliations:** Highway and Airport Engineering Laboratory, Public Works Engineering Department, Faculty of Engineering, Mansoura University, Mansoura, Egypt

**Keywords:** Asphalt binder, Recycled-low density polyethylene, Waste engine oil, Rejuvenation, Morphological, thermal, and chemical characterization, Rheological, and mechanical characterization, Engineering, Civil engineering

## Abstract

This study tackles the environmental challenges associated with the growing accumulation of waste plastic (WP), specifically recycled low-density polyethylene (R-LDPE), by examining its impact on the performance of asphalt binders amid extreme weather and heavy traffic conditions. The research investigates how R-LDPE modifies both virgin asphalt binder (VAB) and recycled asphalt binder (RAB) rejuvenated with 15% waste engine oil (WEO). Three proportions of R-LDPE (2%, 3%, and 4% by weight of VAB) were evaluated, with findings indicating that 2% is the optimal amount for enhancing binder properties. The study involved morphological, thermal, and chemical evaluations using scanning electronic microscope (SEM), energy dispersive X-ray spectroscopy (EDX), Fourier transform infrared spectroscopy (FTIR), and thermogravimetric analysis (TGA). Additionally, comprehensive rheological and mechanical properties were assessed, including tests for penetration, softening point, rotational viscosity (RV), dynamic shear at high and intermediate temperatures, multiple stress creep and recovery (MSCR), and linear amplitude sweep (LAS). The results revealed that R-LDPE incorporation significantly improves the thermal stability, stiffness, and rutting resistance of asphalt binders, with modified RAB outperforming VAB in rutting resistance, while fatigue resistance was optimized with 2% R-LDPE. The MSCR test results further revealed enhanced elastic recovery and reduced creep, indicating superior resistance to repeated loading. The LAS test indicated an improvement in fatigue life and better resistance to crack propagation under cyclic loading. Overall, the use of R-LDPE and WEO enhanced the investigated binder’s durability and performance, making them more suitable for hot climates and high-stress applications.

## Introduction

Asphalt binder is a dark, solid or semi-solid hydrocarbon material, either naturally occurring or derived from petroleum distillation^1–5^. Asphalt, a viscous petroleum derivative, is essential for constructing roads, parking lots, airports, and embankment dams^1,2,5–12^. Asphalt binder consists of high molecular weight hydrocarbon chains, categorized as maltenes (oils, aromatic compounds, and resins) and asphaltenes^9^. Virgin asphalt binder (VAB) is crucial for road construction due to its binding properties, strength, durability, impermeability, and cost-effectiveness^3,13,14^. Classically, asphalt binders can be classified based on penetration rates, as outlined by the American Association of State Highway and Transportation Officials (AASHTO), modern classification systems, such as Superpave performance grading (PG) assess suitability based on performance under diverse temperature and loading conditions^4^. Reclaimed asphalt pavement (RAP), a non-renewable resource, is often stockpiled, crushed, tested, and reintegrated into new asphalt binder at production plants^15–18^. With the rapid advancement of road construction, the demand for asphalt binder is anticipated to rise significantly over the next decade^1,2,19–25^. The rheological properties of asphalt binders are complex, exhibiting both viscous and elastic behaviours depending on loading time and temperature. This viscoelastic nature of asphalt binder influences various aspects of road performance^26,27^, highlighting the urgent need to identify alternative materials that can partially replace fossil asphalt^21,24,26,28,29^. Numerous highway agencies are evaluating the environmental suitability and effectiveness of recycled products for road construction^21,23,30–33^.

Consequently, the reduction in natural resource consumption and the reutilization of waste materials are critical concerns across various research fields^34^. Especially, asphalt modification has become essential to maintain the required performance standards of asphalt mixtures for specific weather and traffic loading conditions^13^, . Recent studies explore the use of waste oil (WO) to rejuvenate and waste plastic (WP) to modify asphalt, supporting global efforts to incorporate recycled materials in pavement construction^13,29,30,34–37^.

Industrial production and daily life generate WO and WP, both posing environmental threats. WO presents environmental risks, while WP contributes to “white pollution” due to its slow degradation. Incinerating WP releases harmful gases, further worsening environmental problems^29^. Annually, the US, EU, UK, and Canada generate about 1.3 million tons of waste cooking oil (WCO)^38^. Both WCO and waste engine oil (WEO), which share compositional similarities with asphalt binders, based on the principle of “like dissolves like,” can potentially modify asphalt^29,39^. Jia et al.^40^. have explored the use of WEO residues as a partial asphalt replacement, while DeDene and You^41,42^ introduced the incorporation of WCO and WEO into recycled asphalt binder (RAB) to enhance RAP performance.

From another perspective, plastics are synthetic, non-biodegradable materials derived mainly from refined crude oil petroleum products^17,43,44^. Their cost-effectiveness, light weight, durability, and ease of processing have led to their widespread use in many industries. Plastics possess beneficial properties, including longevity, water resistance, elasticity, strength, and corrosion resistance, as well as being easy to transport and economically valuable^23,45–48^. However, the large amounts of WP generated poses a significant environmental threat. Land-based WP often pollutes waterways, causing flooding and harming marine ecosystems^23,32,37,47,49,50^. It is estimated that approximately 300 million metric tons of WP are generated annually in the worldwide^49,51^, with the material persisting on Earth for up to 4500 years without degradation^4,23,52,53^. Despite this, only 7% is recycled, about 8% is incinerated, and approximately 30% is recycled in China, with the majority ending up in landfills^1,30,47,49^. Recycled thermoplastic polymers from domestic waste, such as polyethylene terephthalate (PET), high-density polyethylene (HDPE), low-density polyethylene (LDPE), polyvinyl chloride (PVC), polypropylene (PP), and polystyrene (PS), can modify and extend asphalt binder^17,25,26,44,54,55^. Thermosetting plastics and synthetic fibers like multilayer laminates, Teflon, polyurethane foam, Bakelite, polycarbonate, melamine, and nylon are non-recyclable and unsuitable for this application^47,56,57^.

A recent review of plastic waste disposal highlighted that most discarded plastic consists of LDPE^23,49^. Its structure, characterized by numerous side chains, results in lower density, increased flexibility, and weaker intermolecular forces compared to HDPE^4^. As the most widely used polyethylene plastic globally, LDPE, commonly found in sheets, bags, and water sachets, presents a major waste management issue in developing countries lacking adequate collection and recycling systems^23,58,59^. LDPE is a thermoplastic that can be remolded multiple times, with a density ranging from 0.91 to 0.94 g/cm³, a crystalline content of 50–60%, and a melting point of approximately 115 °C^23,59^. Recently, recycling plastics in road construction became an effective approach^23,60^. Researchers have explored using recycled low-density polyethylene (R-LDPE) to modify asphalt binders, improving asphalt mixture performance^4,60^. R-LDPE’s abundance and cost-effectiveness offer benefits like reduced thermal susceptibility, improved deformation resistance, and enhanced low-temperature cracking resistance^1,4,12,17^. However, its low compatibility with asphalt binder can cause phase separation at high temperatures^61^.Roads constructed with plastic-bituminous mixtures can significantly extend pavement life^4^. Highway agencies are increasingly adopting additives in asphalt binder mixtures to improve their characteristics, driven by sustainability and environmental concerns^1,60^.

Addressing the previously identified research gaps is critical for the effective application of R-LDPE in modifying asphalt binders. Improving the process of sustainability and understanding long-term effects are essential, especially regarding manufacturing and use as a modifier.

## Study objectives and scope of work

The primary aim of this study is to systematically evaluate the efficacy of incorporating R-LDPE and WEO as sustainable modifiers for VAB. Specifically, the research seeks to determine whether the combined addition of these environmentally friendly materials can significantly enhance the binder’s morphological, thermal, chemical, rheological, and mechanical properties. The study focuses on assessing improvements in key performance indicators such as rutting resistance, fatigue life, and thermal stability, with the overarching hypothesis that these modifiers can substantially improve binder performance while promoting sustainable pavement practices.

A comprehensive scope aims to establish a scientifically rigorous foundation for the potential application of recycled polymers and waste oils in sustainable road construction practices, as detailed below:

- Material Characterization: Comprehensive analysis of R-LDPE and WEO to determine their compatibility and interaction with VAB.
- Binder Modification and Preparation: Formulation of modified asphalt binders using varying proportions of R-LDPE and WEO to identify optimal additive combinations.
- Laboratory Testing: Evaluation of modified binders through a series of advanced tests, including morphological, thermal, chemical, rheological, and mechanical assessments.
- Performance Evaluation: Investigation of key performance parameters such as rutting resistance, fatigue endurance, and thermal stability to verify the performance enhancements attributable to the modifiers.

## Material selection and sample preparation

In this research, modified asphalt binder with R-LDPE was produced using the following materials:

### Virgin asphalt binder (VAB)

The VAB used in this study has a penetration grade of 60/70, sourced directly from the petroleum refining process. VAB, a key material in asphalt mixtures, plays a crucial role as the primary adhesive, providing cohesion and ensuring the long-term durability of pavement structures under varying traffic and environmental conditions. The 60/70 penetration grade indicates its intermediate stiffness, making it suitable for road construction in regions with moderate climatic conditions. This binder was supplied by Alexandria Specialized Petroleum Products Company (ASPPC), Egypt.

### Recycled asphalt binder (RAB)

The RAB used in this study was sourced from the Benha-Cairo highway, where the pavement had been in service for approximately more than 10 years. A two-step recovery process was implemented to extract the asphalt binder from the RAP: extraction and distillation. During the extraction phase, methylene chloride was used as a solvent to separate the binder from the aggregate. This was followed by a distillation process to remove any remaining solvents, leaving behind the RAB. The entire recovery process adhered to the specifications outlined in ASTM D2172/D2172M-2^62^, ensuring the quality and consistency of the reclaimed binder.

### Recycled-low density polyethylene (R-LDPE)

**Fig. 1:**
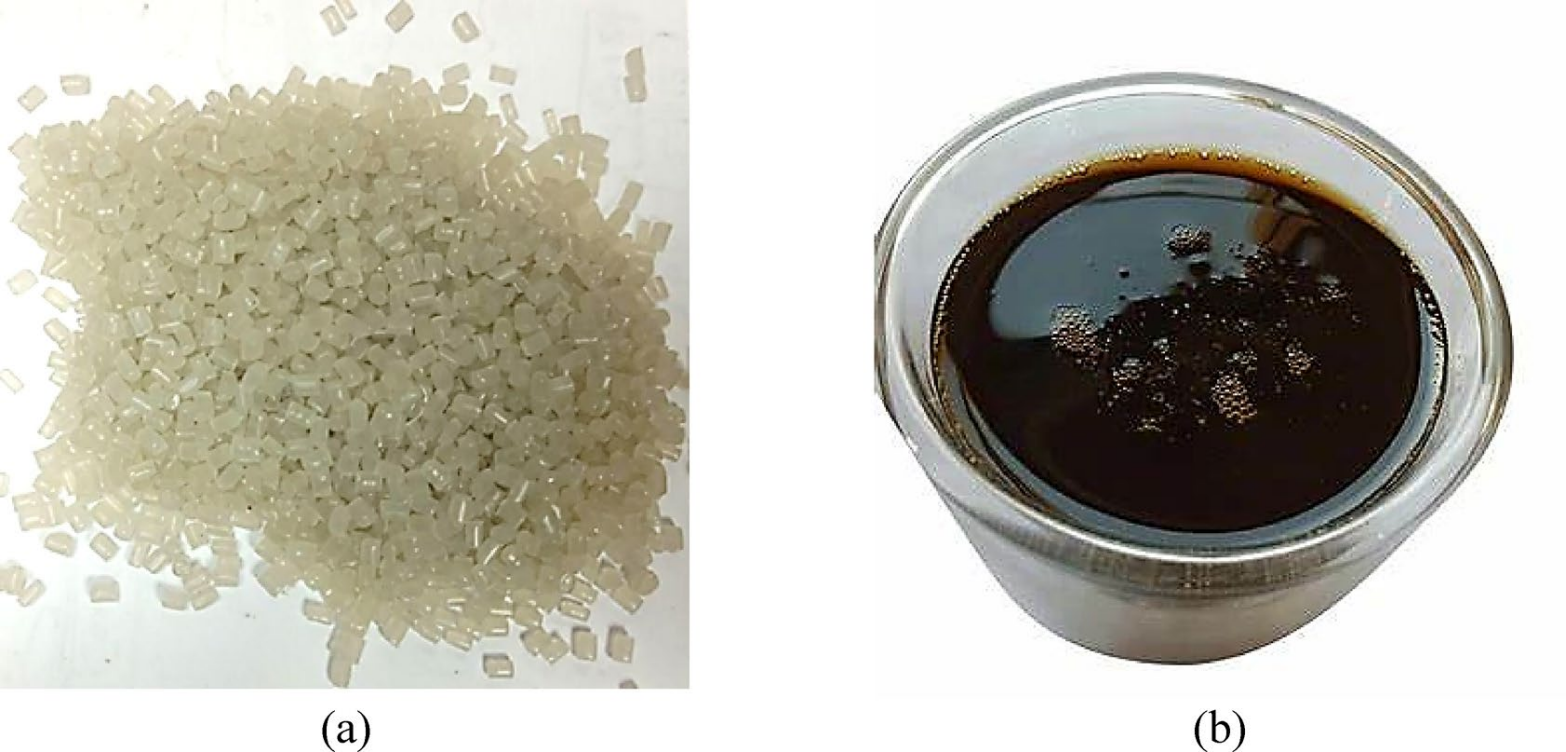
Incorporated recycled materials: (**a**) R-LDPE, and (**b**) WEO.

The R-LDPE (Fig. 1a) used in this study was sourced from the Shuman factory located along Mansoura-Gamasa Road. This material is produced by recycling post-consumer plastic waste, contributing to sustainable practices by reusing discarded plastics. R-LDPE is known for its flexibility, lightweight characteristics, and resistance to impact and moisture, making it an ideal material for various applications, including asphalt modification. Its low density and durable properties enhance the performance of asphalt mixtures, while offering environmental benefits by reducing plastic waste.

### Recycled-low density polyethylene-modified asphalt binder (VAB + R-LDPE)

The R-LDPE-modified asphalt binder was produced by incorporating R-LDPE into VAB at varying proportions of 2%, 3%, and 4% by weight of the VAB. Following established procedures^63^, the mixing speed was maintained above 120 rpm, with temperatures ranging between 160 °C and 170 °C. Previous studies, by Dalhat and Al-Abdul Wahhab, have reported successful blending of R-LDPE with asphalt binder by mixing at 160 °C for 30 min, using a shear speed of approximately 5000 rpm^64^. Similarly, Hameed et al. used a shear mixer at 3000 rpm for 60 min at 170 °C to produce an R-LDPE-modified asphalt binder^65^.

**Fig. 2:**
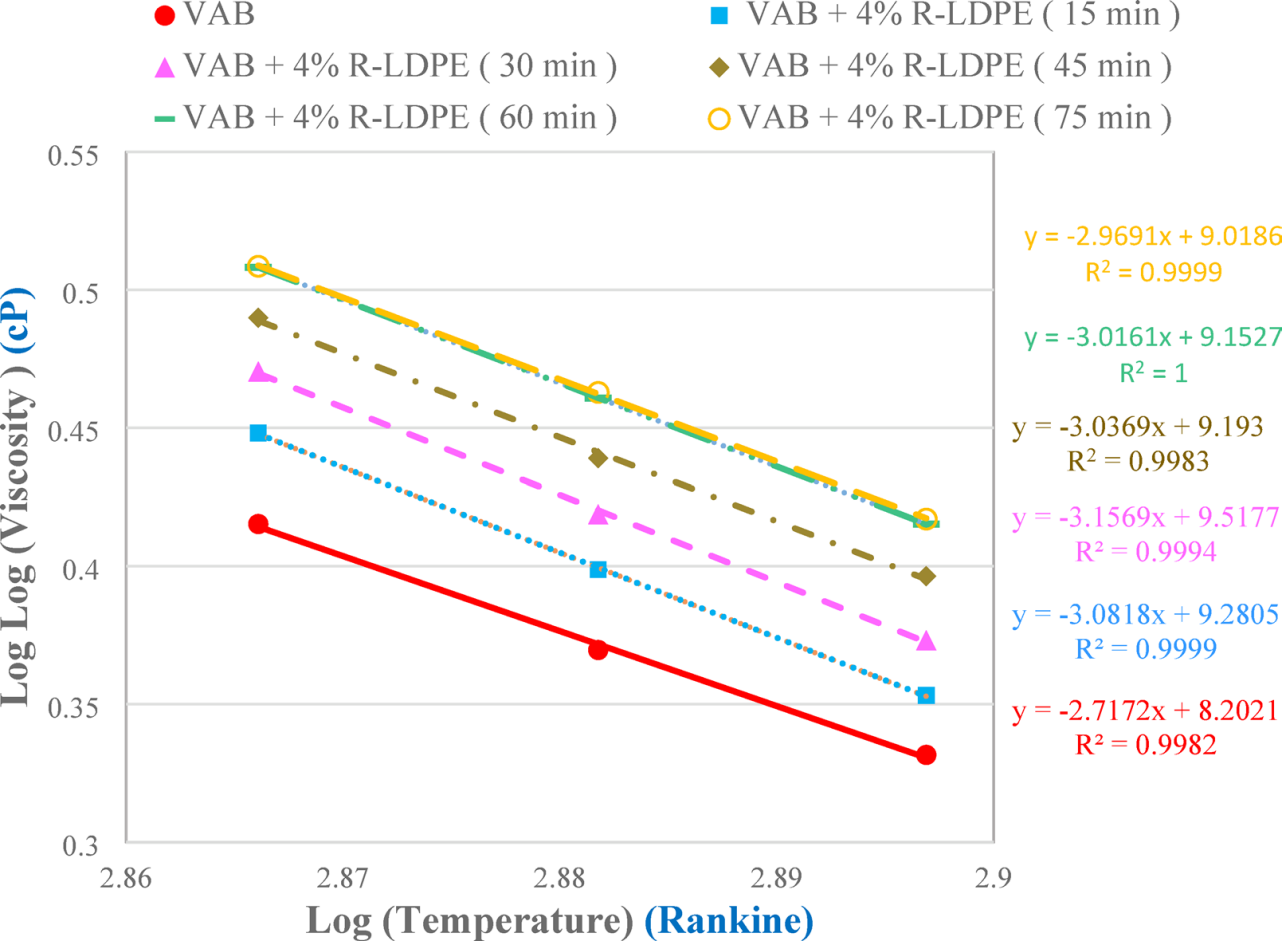
Viscosity-temperature correlations for VAB modified with 4% R-LDPE at varied mixing times.

The optimum mixing time for combining R-LDPE with VAB was determined by conducting the rotational viscosity (RV) test in accordance with ASTM D4402/D4402M-23 standard^66^. Figure 2 demonstrates the relationships between viscosity (in cP) and temperature (in Rankine) for the VAB modified with 4% R-LDPE at varied mixing times, providing insight into the viscosity behavior and processing characteristics of the modified binder.

During the mixing process, a locally manufactured high-shear mixer was employed, operating at speeds between 3000 and 5000 rpm. As shown in Fig. 2, the viscosity of the VAB modified with 4% R-LDPE blend stabilized after 60 min at testing temperatures of 135 °C, 150 °C, and 165 °C. A mixing temperature of 160 °C was selected for the production of VAB modified with R-LDPE, based on supporting results from the literature^63–65,67^. Consequently, the optimal mixing parameters for local trials were determined to be a constant shear speed of 3000 rpm at 160 °C for 60 min to achieve a uniformly modified asphalt binder.

Previous studies have also recommended a mixing time of 60 min^65^, while ASTM and California department of transportation (Caltrans) guidelines suggested extending the mixing time beyond 45 min to ensure thorough modification.

### WEO-rejuvenated R-LDPE-modified asphalt binders (RAB + 15% WEO + 2% R-LDPE)

This study employed recycling tractor WEO LDPE (Fig. 1b) technology, utilizing oil collected from the lubrication systems of agricultural machinery, which require regular oil changes as part of routine maintenance. A dosage of 15% WEO by weight of the RAB was identified as optimal, based on morphological, chemical, and thermal evaluations. This dosage effectively restored the aged binder’s properties and met the performance criteria of Egyptian standards, including a minimum softening point of 45 °C, penetration grade 60/70, and PG 64-XX classification, confirming its suitability for pavement applications. To enhance high-temperature performance, 2% R-LDPE was incorporated into the rejuvenated binder, resulting in a PG 76-XX grade suitable for hot climates in southern Egypt. The WEO-rejuvenated RAB was first prepared with 15% WEO in the specified ratios using a high-shear mixer at 135 °C and 1500 rpm for 30 min. Then, it was followed by the addition of 2% R-LDPE at 160 °C and 3000 rpm for 60 min, calculated based on the total weight of the rejuvenated binder. The blend, RAB + 15% WEO + 2% R-LDPE, was produced under consistent mixing conditions to ensure uniformity. The selection of 2% R-LDPE was guided by its ability to meet the performance requirements outlined in Sect. 5.

In addition, the potential release of volatile components from the WEO–RAB blend at elevated temperatures was considered during the blending process. To minimize volatilization, the mixing temperature was carefully controlled and maintained within the range typically recommended in literature reviews for WEO incorporation, ensuring sufficient blending without excessive thermal degradation. Furthermore, the short-term aging behavior of the prepared WEO–RAB binders was evaluated using the RTFO procedure^75^, which provides insights into the thermal stability and volatility of the material under controlled heating and air exposure. The RTFO test allows for assessing the resistance of the binder to mass loss and oxidative hardening, thereby offering an indirect measure of the binder’s stability during mixing and construction processes.

## Research methodology

Extensive testing was conducted on VAB, R-LDPE-modified VAB, and WEO-rejuvenated RAB with R-LDPE modification to evaluate their morphological, thermal, chemical, rheological, and mechanical characteristics. the following tests were carried out:

1. Morphological, Chemical, and Thermal Characterization: The surface morphology, thermal stability, and chemical compositions of R-LDPE and the modified asphalt binders were analyzed using Scanning electronic microscope (SEM)^68^, Energy dispersive X-ray spectroscopy (EDX)^69^, Fourier Transform Infrared Spectroscopy (FTIR)^70^, and Thermogravimetric Analysis (TGA)^71^. These tests aimed to assess the surface topography, elemental composition, functional groups, degradation behavior, and interaction mechanisms of R-LDPE and WEO within the asphalt matrix.
2. Rheological and Mechanical Characterization: A comprehensive suite of tests was conducted to evaluate the mechanical and rheological performance of the asphalt binders:
  Penetration and Softening Point Tests, following ASTM standards^72,73^, were used to determine binder consistency and temperature sensitivity, including the calculation of the penetration index (PI).
  RV was measured at various temperatures; RV^66^ was used to evaluate the workability and flow characteristics of the asphalt binders.
  Dynamic Shear Rheometer (DSR) tests^74^ were performed to determine the rutting and fatigue parameters of the asphalt binders under original, rolling thin film oven (RTFO)^75^, and pressure aging vessel (PAV)^77^-aged conditions. The binder complex shear modulus (G*) and binder phase angle (δ)) were determined at different temperatures. Then, rutting performance was assessed by measuring the (G*/sin δ) parameter, while fatigue resistance was evaluated using the (G*sin δ) parameter.
  Multiple Stress Creep and Recovery (MSCR) test^76^ was employed to assess the elastic and non-elastic responses of the modified binders under different stress levels. The percent recovery (*%R*) and non-recoverable creep compliance (*J*_*nr*_) were measured to determine the rutting resistance of the investigated binders.
  Linear Amplitude Sweep (LAS) test^78^ was conducted to evaluate the fatigue resistance of asphalt binders at intermediate temperatures. The fatigue life (*N*_*f*_) and undamaged parameter (*α*) were calculated based on the simplified viscoelastic continuum damage (S-VECD) theory, providing insights into the durability and crack resistance of the modified binders.

**Fig. 3:**
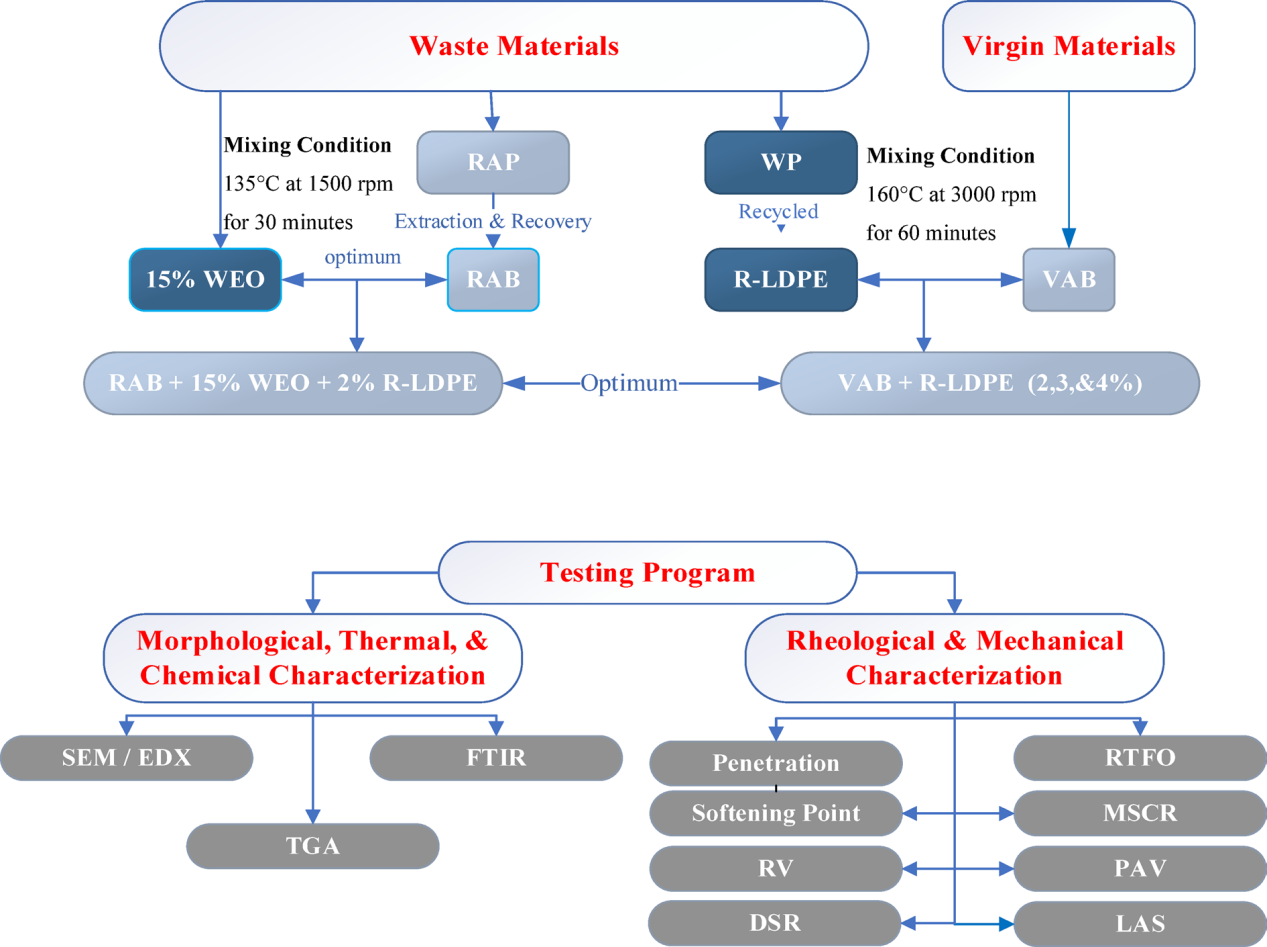
Flowchart of the investigated materials & testing methodology.

To maintain consistency in the production process, each test result was averaged from three individual tests to ensure accuracy and enable reliable comparative analysis. The testing program covered a comprehensive range of assessments and following the American society for testing and materials (ASTM) standards, as shown in Fig. 3.

## Results and discussion

### Morphological, thermal, & chemical characterization

#### Scanning electronic microscope (SEM) / Energy dispersive X-ray spectroscopy (EDX)

**Fig. 4:**
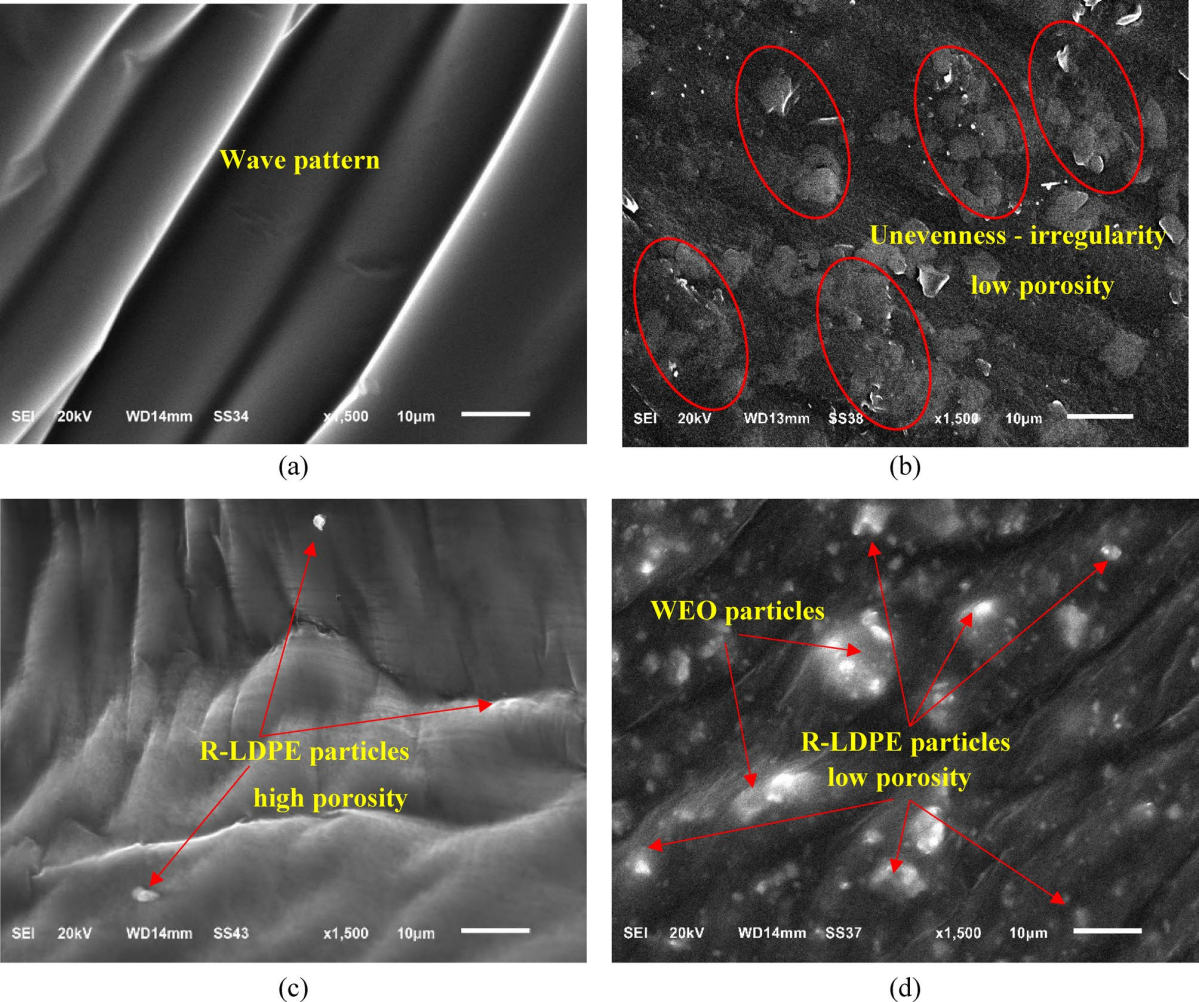
SEM images of the investigated asphalt binders with optimum dosages of modifiers and rejuvenators: (**a**) VAB; (**b**) R-LDPE; (**c**) R-LDPE-modified VAB; and (**d**) WEO-rejuvenated RAB and modified with R-LDPE.

SEM and EDX analyses are crucial in understanding the surface morphology, structure, physical properties, and elemental composition of R-LDPE and modified asphalt binders containing R-LDPE. For this study, a JEOL JSM 65101v microscope was employed for SEM imaging^68^, while an Oxford X-Max 20 apparatus was used for EDX analysis^69^. Figure 4 presents the SEM images, while Table 1 details the corresponding EDX results, providing a thorough examination of R-LDPE and its influence on different types of asphalt binders, such as VAB, R-LDPE-modified VAB, and WEO-rejuvenated RAB with R-LDPE modification.

The SEM analysis was performed on all modified binders in their original form, with a magnification of ×1500 and an operating voltage of 20 kV, as shown in Fig. 4. The surface analysis of the asphalt binders and modifiers revealed distinct characteristics. The surface of the VAB was smooth and consistent, showing no significant irregularities or low porosity, indicating a uniform material structure (Fig. 4a).

In contrast, the surface of the R-LDPE exhibited notable roughness and unevenness, with a high degree of irregularity and high porosity due to the presence of impurities and the degradation of polymer chains during the recycling process, leading to the formation of voids and gaps within the material swelling (Fig. 4b), consistent with typical post-consumer plastic textures^48,57^.

When R-LDPE was introduced into the VAB, the particles appeared mostly spherical with blurry edges due to swelling (Fig. 4c), suggesting some interaction between the binder and the R-LDPE. The swelling phenomenon can be attributed to the incomplete dissolution of the plastic particles in the binder, resulting in the absorption of a portion of the asphalt binder and the subsequent expansion of the particles, as the R-LDPE did not fully melt or integrate with the binder^48,57^.

When R-LDPE was introduced into the VAB, the particles appeared mostly spherical with blurred edges due to swelling (Fig. 4c), indicating partial interaction between R-LDPE and the surrounding binder. This swelling is attributed to the partial dissolution of plastic particles, which absorb a portion of the binder and expand, as R-LDPE does not fully melt or blend homogeneously^48,57^. The resulting rougher surface morphology positively contribute to the binder’s resistance to deformation, improve load transfer capabilities, and enhance overall durability. However, these benefits should be carefully balanced to avoid adverse effects such as excessive stiffness or brittleness, which could compromise long-term performance.

Similar observations were made in the WEO-rejuvenated RAB, where strong bonding between R-LDPE and the binder matrix was evident (Fig. 4d), indicating enhanced interaction and improved performance of the modified binder.

These SEM observations suggest potential interactions between R-LDPE and the asphalt binder at the microstructural level, which may influence the binder’s properties. The addition of 2% R-LDPE to both VAB and WEO-rejuvenated RAB resulted in improved rheological properties at high temperatures, contributing to better road surface performance and addressing the challenge of plastic waste disposal, as reported in literature^57^.

According to the presented results in Table 1, the elemental composition of the various binders provides valuable insights into their structural differences and how these affect their performance. The elemental composition of the VAB revealed a high carbon (C) content of 91.19%, with sulfur (S) at 7.46% and oxygen (O) at 1.36%, typical of petroleum-derived materials and indicative of strong adhesive and elastic properties. In addition, R-LDPE exhibited a significant C content at 80.21%, with increased O (6.56%) and trace metals like nitrogen (N) (00.65%) levels, reflecting its plastic waste origin and the nature of the polymers involved. The RAB composition was more similar to VAB, with C at 87.56%, S at 4.30%, and O at 6.34%. However, RAB also contained trace metals like aluminum (Al), silicon (Si), iron (Fe), and calcium (Ca), likely from road aggregate and environmental exposure during its decade of service.

The incorporation of R-LDPE into both VAB and WEO-rejuvenated RAB resulted in significant changes in their elemental composition. In VAB modified with 2% R-LDPE, there was a slight decrease in N content (0.59%), attributed to the R-LDPE inclusion, while decrease in C, S and increase in O levels. This indicates moderate interaction between the R-LDPE and VAB. In contrast, RAB rejuvenated with 15% WEO and modified with 2% R-LDPE showed substantial changes. N content reduced sharply to 0.3%, while C dropped to 81,20%, and S decreased to 4,5%. These shifts suggest a stronger modification effect from the combined WEO and R-LDPE, significantly altering the RAB’s structure. The increased O content (9.95%) also highlights the enhanced oxidative potential of this modified binder.

The variations in chemical composition, particularly the decreased N and increased O in the modified binders, are indicative of enhanced performance properties such as improved flexibility and resistance to environmental stresses, making the modified binders suitable for long-lasting pavement applications^48,57^.

**Table 1:** EDX results of the investigated asphalt binders with optimum dosages of modifiers and rejuvenators.

VAB		RAB		*R*-LDPE		VAB + 2% *R*-LDPE		RAB + 15% WEO + 2% *R*-LDPE	
Element	Weight%	Element	Weight%	Element	Weight%	Element	Weight%	Element	Weight%
C	91.19	C	87.56	C	80.21	C	87.99	C	81.20
S	7.46	S	4.30	S	0.30	S	5.39	S	4.50
O	1.36	O	6.34	O	6.56	O	4.91	O	3.65
		Al	0.12	N	0.65	N	0.59	N	0.30
		Si	0.42						
		Fe	0.27						
		Ca	0.99						

#### Fourier transform infrared spectroscopy (FTIR)

FTIR spectroscopy tests^70^ were conducted to analyze the functional groups present in the materials and to observe how these groups interact within the modified asphalt binders. The tests employed a JASCO FT/IR6800 spectrometer, operating in the range of 4000 to 400 cm^− 1^ with a resolution of 4 cm^− 1^. The setup involved using a thin potassium bromide (KBr) disk, where the asphalt binder mixture was spread, allowing for the detection of molecular vibrations. The spectroscopic analysis was carried out using a diamond reflective attachment, which captured the spectra of the asphalt binder mixtures under controlled environmental conditions.

**Fig. 5:**
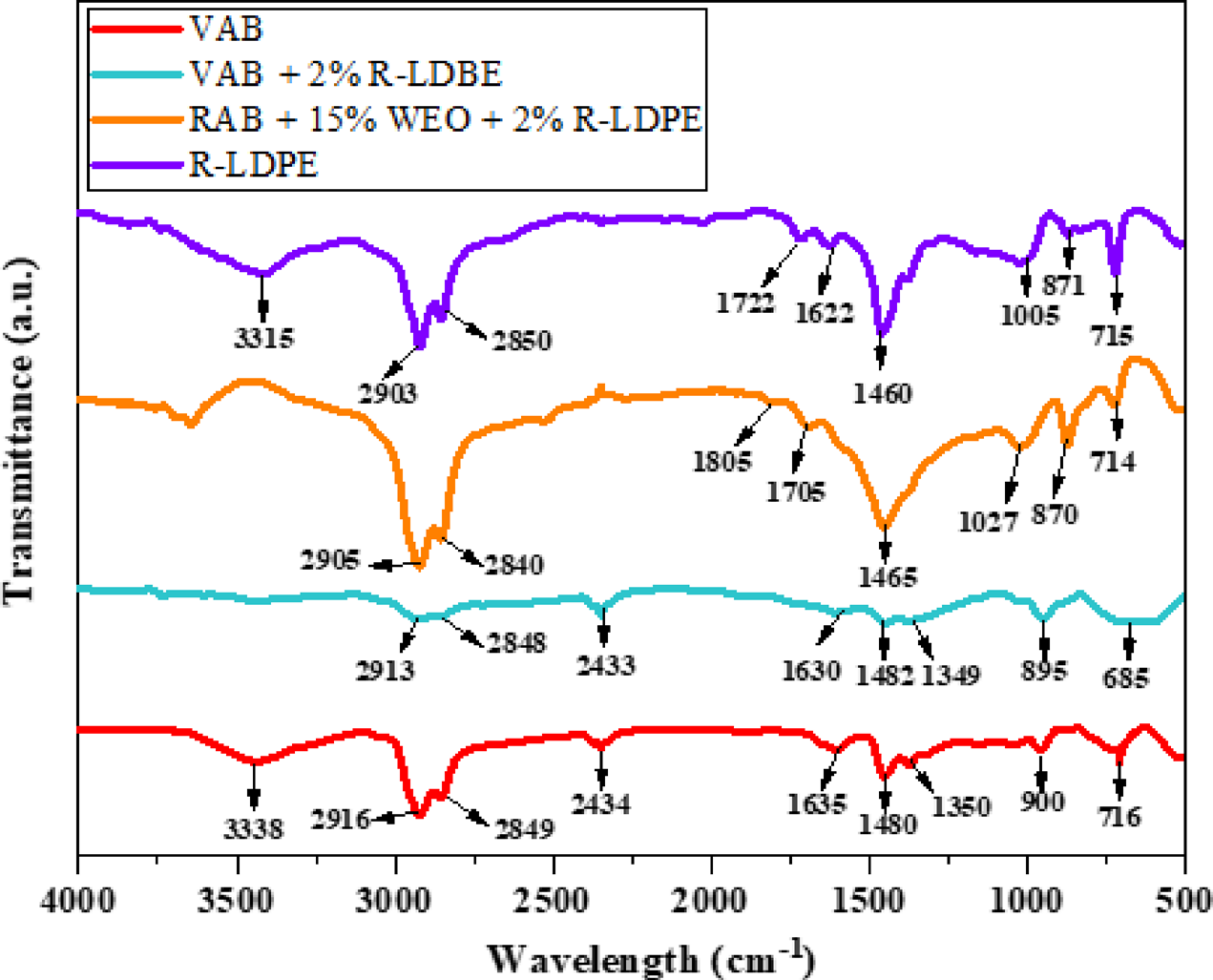
FTIR spectrum of the investigated asphalt binders with optimum dosages of modifiers and rejuvenators.

The FTIR spectra (Fig. 5) for VAB, R-LDPE, and 2% R-LDPE-modified asphalt binders revealed distinct absorption bands corresponding to key functional groups, as summarized in Table 2. In the VAB spectrum, two major peaks at 2916 cm⁻¹ and 2849 cm⁻¹ were attributed to aliphatic C–H stretching vibrations, while a broad band at 3338 cm⁻¹ corresponded to O–H stretching, indicating the presence of hydroxyl groups. Additional bands at 900 cm⁻¹ and 716 cm⁻¹ were related to C–H bending in hydrocarbons^7^. The presence of carbonyl groups (C = O) was identified by a peak at 1635 cm⁻¹, and peaks at 1480 cm⁻¹ and 1350 cm⁻¹ suggested the presence of unsaturated hydrocarbons due to C = C and C–H deformation vibrations, respectively.

The R-LDPE spectrum displayed a similar C–H stretching pattern with absorption bands at 2903 cm⁻¹ and 2850 cm⁻¹. A noticeable O–H band at 3315 cm⁻¹ indicated hydroxyl-containing species. Peaks at 1705 cm⁻¹ and 1622 cm⁻¹ confirmed the presence of carbonyl compounds, and a distinct band at 1005 cm⁻¹ was attributed to sulfur oxide (S = O) functional groups^34,63^. Additionally, bands at 871 cm⁻¹ and 715 cm⁻¹ were associated with C–H bending, as shown in Table 2.

When R-LDPE was incorporated into both VAB and WEO-rejuvenated RAB, notable spectral changes occurred. The intensification of C = O stretching bands at 1805 cm⁻¹ and 1700 cm⁻¹, along with the enhanced S = O band at 1027 cm⁻¹, indicated increased contributions of oxygenated and sulfur-containing compounds—likely originating from oxidative interactions involving R-LDPE and residual components in WEO. Interestingly, the O–H stretching band observed in the VAB spectrum disappeared in the modified binders, suggesting that hydroxyl groups were either consumed during chemical interactions or replaced by more stable functional groups^34,63^. These spectral changes reflect molecular-level modifications, including possible hydrogen bonding, esterification, or oxidation, which contribute to the enhanced compatibility and stability of the modified asphalt binders. The observed chemical transformations highlight the synergistic effect of R-LDPE and WEO in altering the binder’s microstructure and improving its performance characteristics.

**Table 2:** FTIR wavelength analysis and their functional groups of the investigated asphalt binders with optimum dosages of modifiers and rejuvenators.

VAB		VAB + 2% *R*-LDPE	
Wavelength $\:\left({\text{c}\text{m}}^{-1}\right)$	Functional group	Wavelength $\:\left({\text{c}\text{m}}^{-1}\right)$	Functional group
3338	- OH stretching	–	- OH stretching
2916	CH-Alkane stretching	2913	CH-Alkane stretching
2849	C-H	2847	C-H
2434	O–H stretch	–	O–H stretch
1635	C = O stretch	–	C = O stretch
1480	C = C stretching	1482	C = C stretching
1350	C–H	–	C–H
900	C–H bending	895	C–H bending
716	C-H bending	685	C-H bending

RAB + 15% WEO + 2% R-LDPE		R-LDPE	
Wavelength$\:\left({\text{c}\text{m}}^{-1}\right)$	Functional Group	Wavelength$\:\left({\text{c}\text{m}}^{-1}\right)$	Functional Group
–	-OH stretching	3315	-OH stretching
2905	CH-Alkane stretching	2903	CH-Alkane stretching
2861	C-H	2850	C-H
1805 − 1700	C = O	1705 − 1622	C = O
1465	C = C stretching	1460	C = C stretching
1027	S = O	1005	S = O
870	C–H bending	871	C–H bending
714	C-H bending	715	C-H bending

#### Thermogravimetric analysis (TGA)

TGA was conducted to assess the thermal stability and weight loss behavior of R-LDPE as a function of temperature. A TGA-50 instrument was used for this purpose^71^, with the following test parameters: a flow of high-purity nitrogen was maintained at a rate of 20 mL/min, and the R-LDPE sample, weighing between 3 and 10 mg, was gradually heated at a rate of 15 °C per minute up to a maximum temperature of 800 °C.

The purpose of the TGA test was to determine the temperatures at which significant weight loss occurs, reflecting the material’s decomposition profile and thermal stability. The testing for R-LDPE and WEO results revealed a residual mass (Mr) of 4.1% and 13.88% and the temperature at which mass loss occurred (T%) was 453.68℃ and 296.94℃, respectively.

**Fig. 6:**
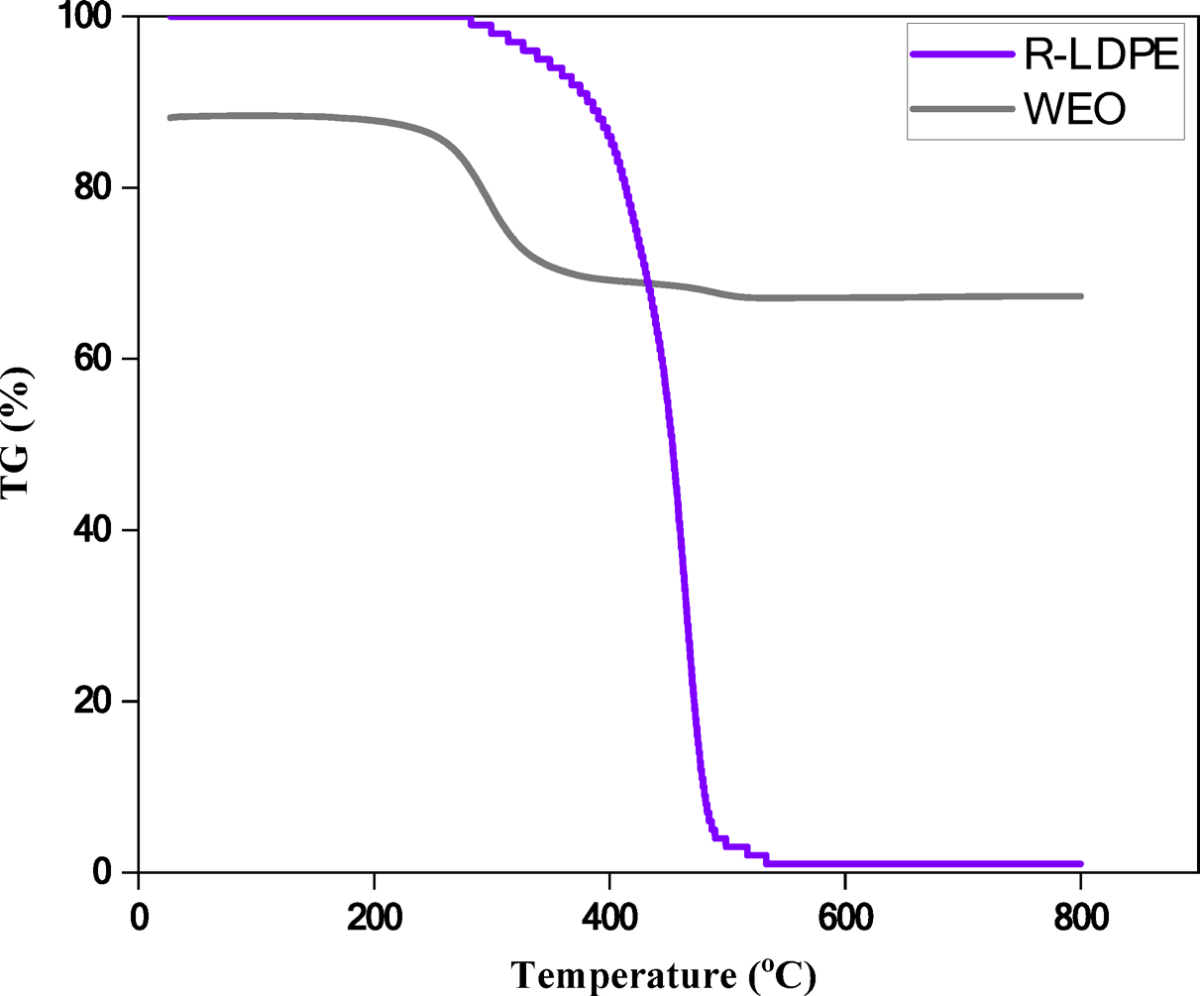
TGA analysis of R-LDPE.

This research conducted a graphical analysis of R-LDPE using TGA, as shown in Fig. 6. The weight loss of R-LDPE occurred in four distinct stages: drying, fast degradation, slow degradation, and a final stage. In drying stage (40 °C to 380 °C), this initial phase involved the volatilization of volatile compounds, including the removal of moisture, solvents, and aromatics, along with the decomposition of highly volatile compounds. In fast degradation stage (380 °C to 500 °C), the material underwent rapid degradation, as seen by a sharp decline in the TGA curve. This stage is marked by a significant breakdown of aromatics, resins, and asphaltenes, leading to the conversion of R-LDPE into volatile compounds. While slow degradation stage (500 °C to 750 °C) exhibited the highest rate of mass loss, primarily due to the thermal breakdown of the remaining material. The degradation process slowed, with contributions from the decomposition of resins and some residual aromatics. The TGA curve showed a gradual reduction in mass, indicating a deceleration of the degradation process. This was primarily due to the thermal breakdown of remaining resins and some residual aromatics, which decomposed at lower rates compared to earlier stages. After the slow degradation phase and during the final stage (above 750 °C), the remaining solid residue could not be physically evaporated or thermally cracked in the TGA analysis. This residue consisted of non-volatile components that remained stable at high temperatures. These stages highlight the thermal behavior of R-LDPE and its degradation pattern, which is critical for understanding its performance as an asphalt modifier, especially under varying thermal conditions. While the TGA result indicate that WEO has significantly lower thermal stability compared to R-LDPE.

The TGA curve showed a gradual reduction in mass, indicating a deceleration of the degradation process. This was primarily due to the thermal breakdown of remaining resins and some residual aromatics, which decomposed at lower rates compared to earlier stages.

### Rheological & mechanical characterization

#### Penetration and softening point

Penetration and softening point tests were conducted in accordance with ASTM D5/D5M-20^72^ and ASTM D36/D36M-14^73^ specifications, respectively. The penetration test evaluated the consistency of the asphalt binder by measuring the depth (in tenths of a millimeter) that a needle penetrates the binder at a temperature of 25 °C. This test is crucial in determining the binder’s hardness and its ability to resist deformation under load. While the softening point test measured the temperature at which the asphalt binder begins to flow, indicating its thermal susceptibility. This test provides insight into the binder’s performance at elevated service temperatures.

**Fig. 7:**
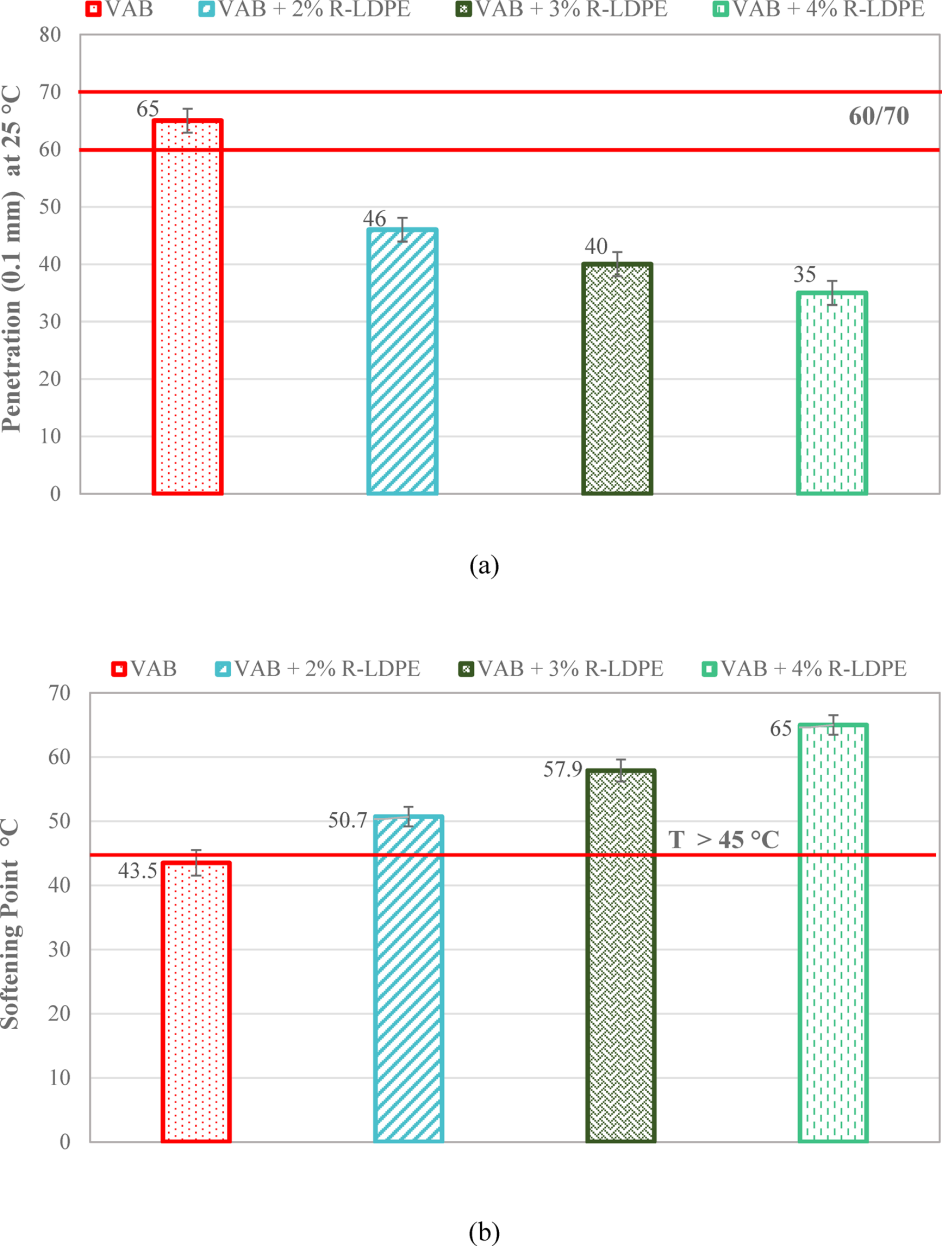

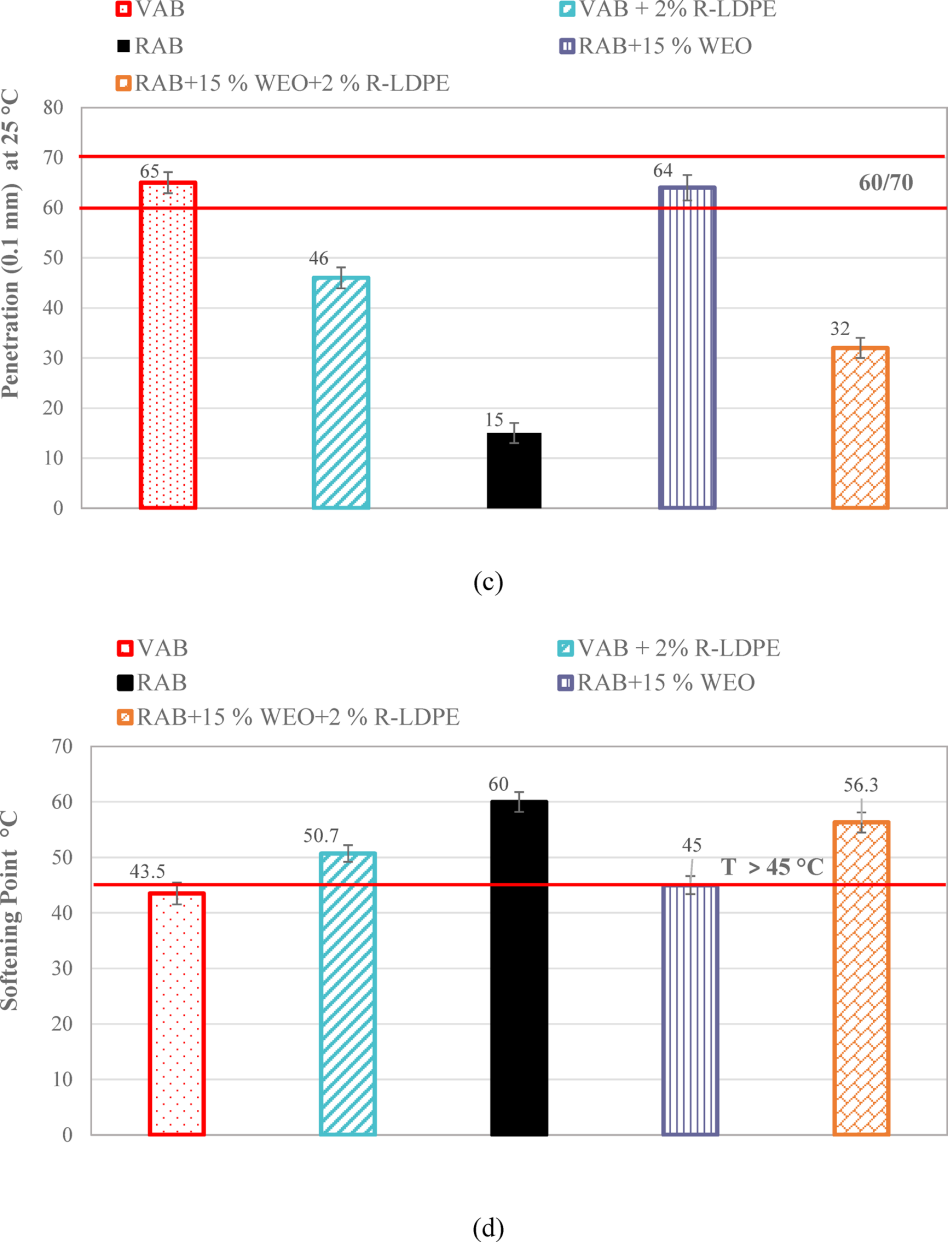
Penetration and softening point results of the investigated asphalt binders with optimum dosages of modifiers and rejuvenators.

Figure 7 presents the penetration values and softening point temperatures for asphalt binders modified with R-LDPE. These values were used to calculate the PI, a parameter that reflects the temperature sensitivity of the binder. A higher PI indicates improved resistance to temperature fluctuations and a more stable asphalt binder across varying climatic conditions^9^.

The addition of R-LDPE to the asphalt binder significantly impacted the penetration and softening point values^47,64,65,67^, as shown in Fig. 7a,b. VAB, used as the base binder, exhibited the highest penetration and the lowest softening point among the binders tested. However, when VAB was modified with 2%, 3%, and 4% R-LDPE by weight, a consistent decrease in penetration and an increase in softening point were observed. This indicates a progressive enhancement in the stiffness and thermal stability of the asphalt binder with increasing R-LDPE content. The swelling of R-LDPE particles in the VAB matrix contributed to maltene incorporation, leading to stronger interactions between the polar asphaltene molecules and R-LDPE. This interaction increased the elasticity of the binder, which is supported by higher levels of carbon and asphaltene content^19,26,63–65,67,79^.

A one-way ANOVA test was conducted to evaluate the statistical significance of the differences in penetration and softening point values between the VAB and the modified binder with 2% R-LDPE. The results of penetration indicated a significant effect of the modification, as evidenced by an F (Fisher’s ratio) -value of 7.71, which is less than the F _*critical*_ -value of 138.46, and a corresponding P-*value* of 0.0003 < 0.05. Similarly, the analysis of the softening point revealed a highly significant effect an F _*critical*_ -value of 104.89, and a P-value of 0.0005.

The F _*critical*_ -value of 138.46 and 104.89 for both the penetration and the softening point of the asphalt binders. supports the presence of a notable effect in the asphalt binder results. These findings confirm that that adding 2% R-LDPE to VAB significantly enhances binder performance, as demonstrated by statistically significant differences between the VAB and VAB + 2% R-LDPE groups.

On the other hand, The RAB showed lower penetration values and higher softening points compared to the asphalt binders studied, including VAB. Notably, the incorporation of EO to RAB resulted in a steady increase in penetration values and a corresponding decrease in softening point, attributed to its lower viscosity. Rejuvenating RAB with 15% WEO produced a penetration grade of 60/70 and met the Egyptian standards for a minimum softening point of 45 °C. This rejuvenated RAB had penetration and softening point values comparable to VAB. When 2% R-LDPE was added to the rejuvenated binder (RAB + 15% WEO), Fig. 7c,d show that the modified binder had lower penetration, and a higher softening point compared to both VAB and VAB + 2% R-LDPE.

These findings indicate that the addition of 15% EO and 2% LDBE to RAP produces statistically significant improvements in both penetration and softening point compared to the original RAP. For penetration and softening point, the analysis revealed a significant effect of the modification, with F _*critical*_ -value of 108.375 and 119, respectively, both greater than the F -value of 7.7086. The corresponding P-values of 0.0005 and 0.0003 were also below the significance level of 0.05, confirming the statistical significance of the improvements.

These results highlight that incorporating R-LDPE into rejuvenated asphalt binders resulted in a more stable and elastic binder, which is expected to improve rutting resistance due to the formation of a polymer network. The R-LDPE-modified binder offered enhanced stiffness, which is favorable for preventing permanent deformation under heavy traffic loads^19,26,63–65,67,79^.

##### Penetration index (PI)

**Fig. 8:**
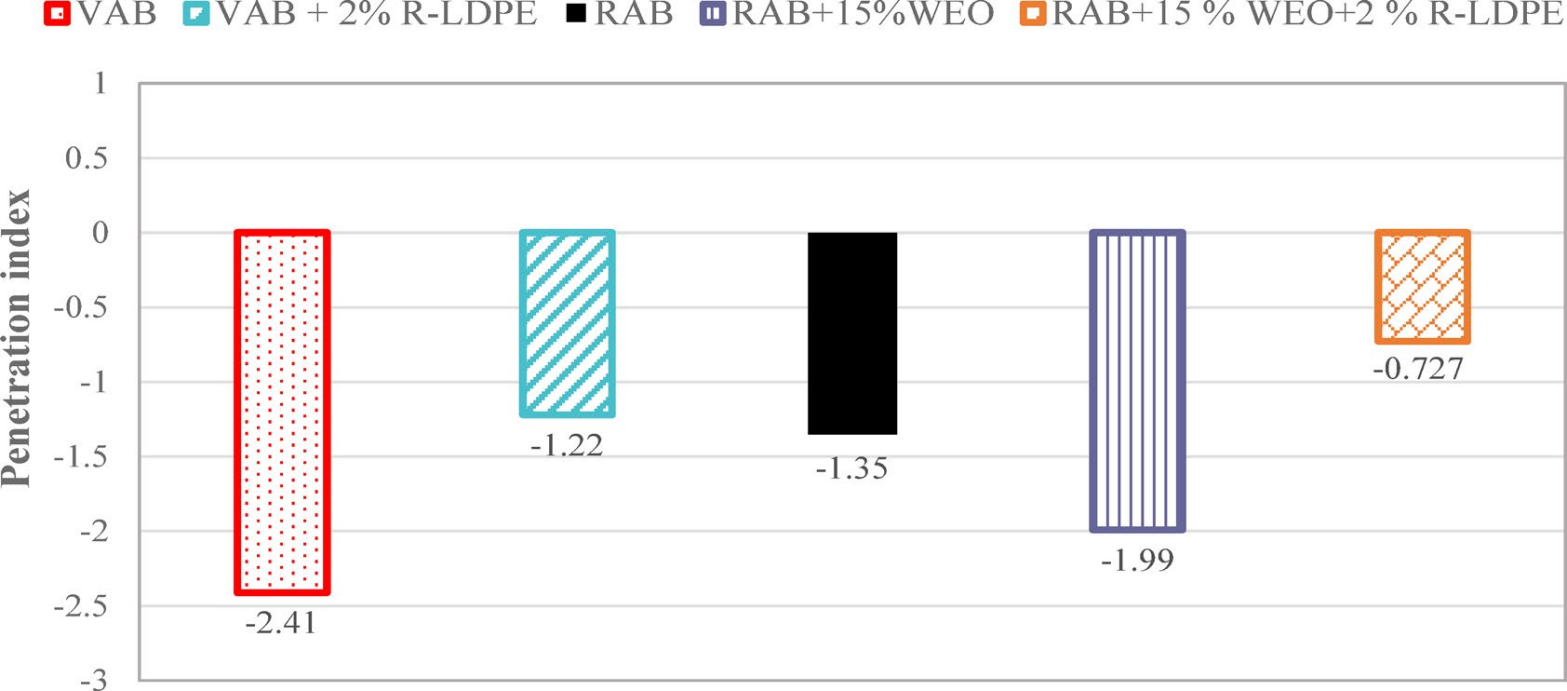
Penetration index results of the investigated asphalt binders with optimum dosages of modifiers and rejuvenators.

The PI analysis in Fig. 8 highlights how the use of R-LDPE as a modifier reduces the temperature susceptibility of asphalt binders. Both VAB and 15% WEO-rejuvenated RAB with R-LDPE modifiers exhibit significantly lower susceptibility to temperature fluctuations than unmodified VAB. This suggests that R-LDPE helps improve the thermal performance of the binder.

Higher PI values indicate binders that are more suitable for hot climates, as they demonstrate less temperature-induced softening. Conversely, lower PI values are preferred for cold climates, as they indicate greater flexibility and resistance to cracking at low temperatures. The results indicate that the R-LDPE-modified binders fall within the optimal range for reducing temperature sensitivity.

Asphalt binders typically have PI values between − 1 and + 1. Those with PI values below − 2 are highly susceptible to temperature variations and tend to become brittle in colder conditions^19,65^. The equation used to calculate the PI from penetration and softening point results, as referenced from^65^, was applied to evaluate the binders’ temperature sensitivity.

#### Rotational viscosity (RV)

**Fig. 9:**
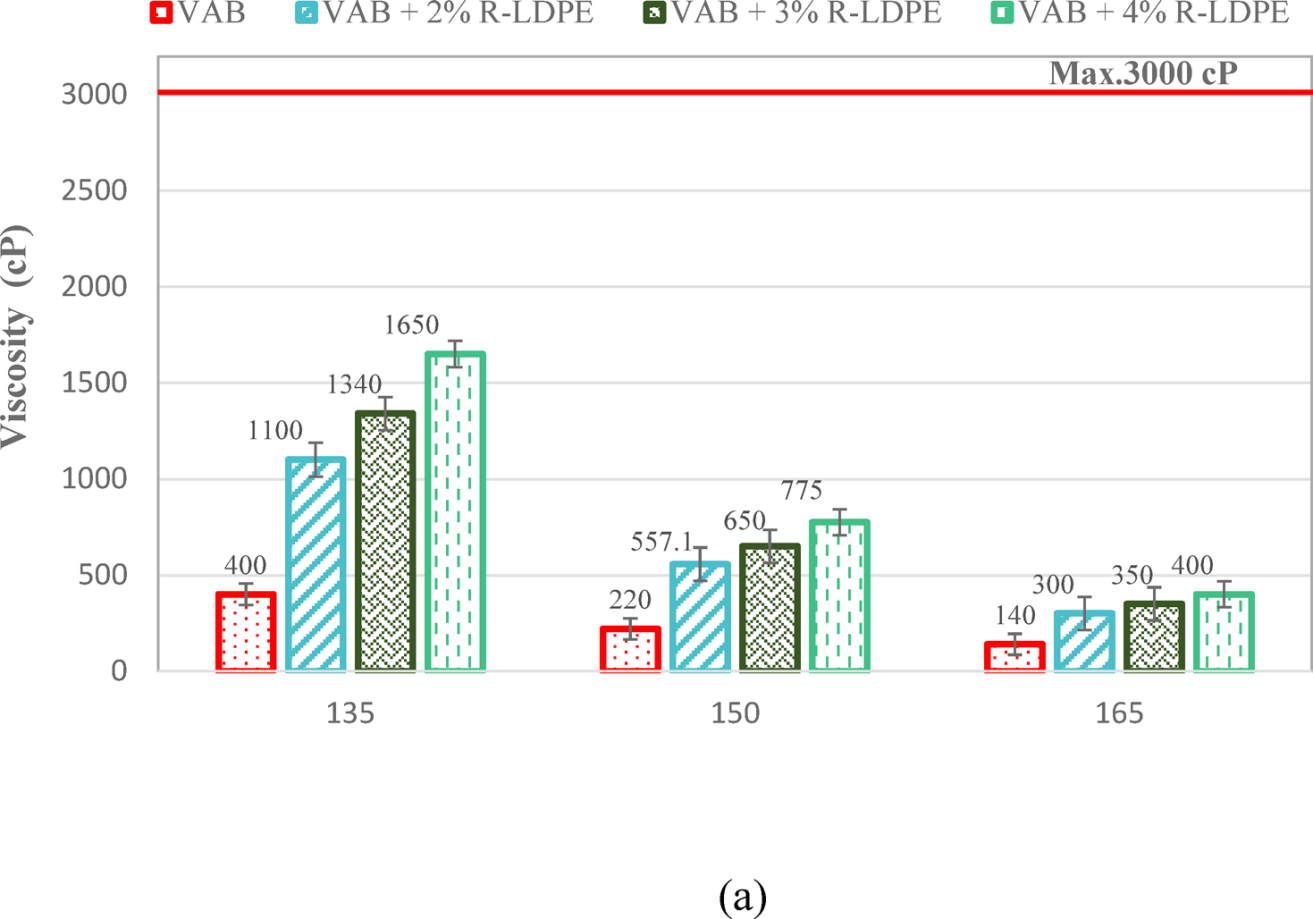

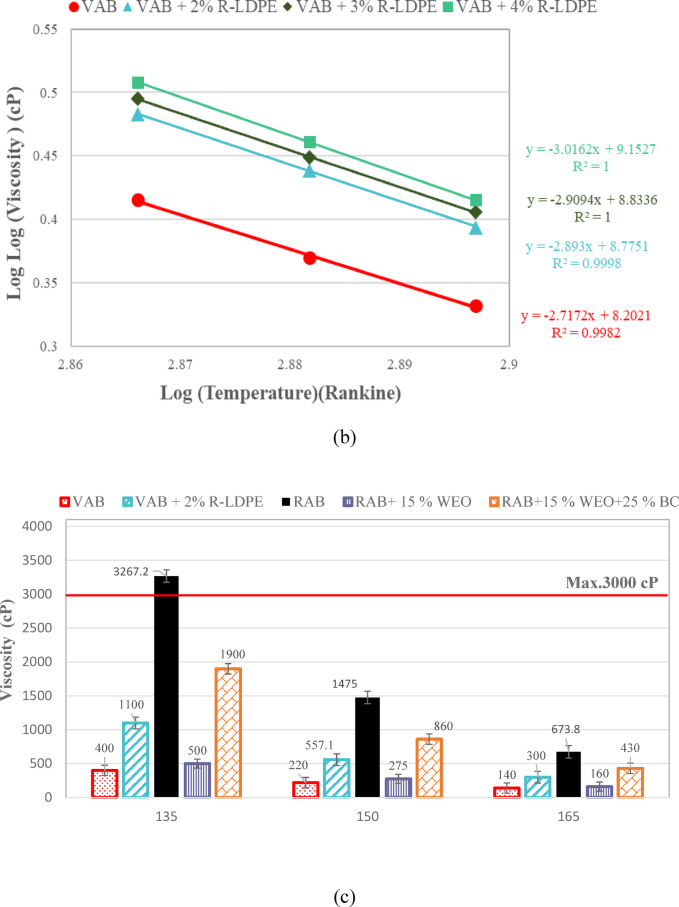

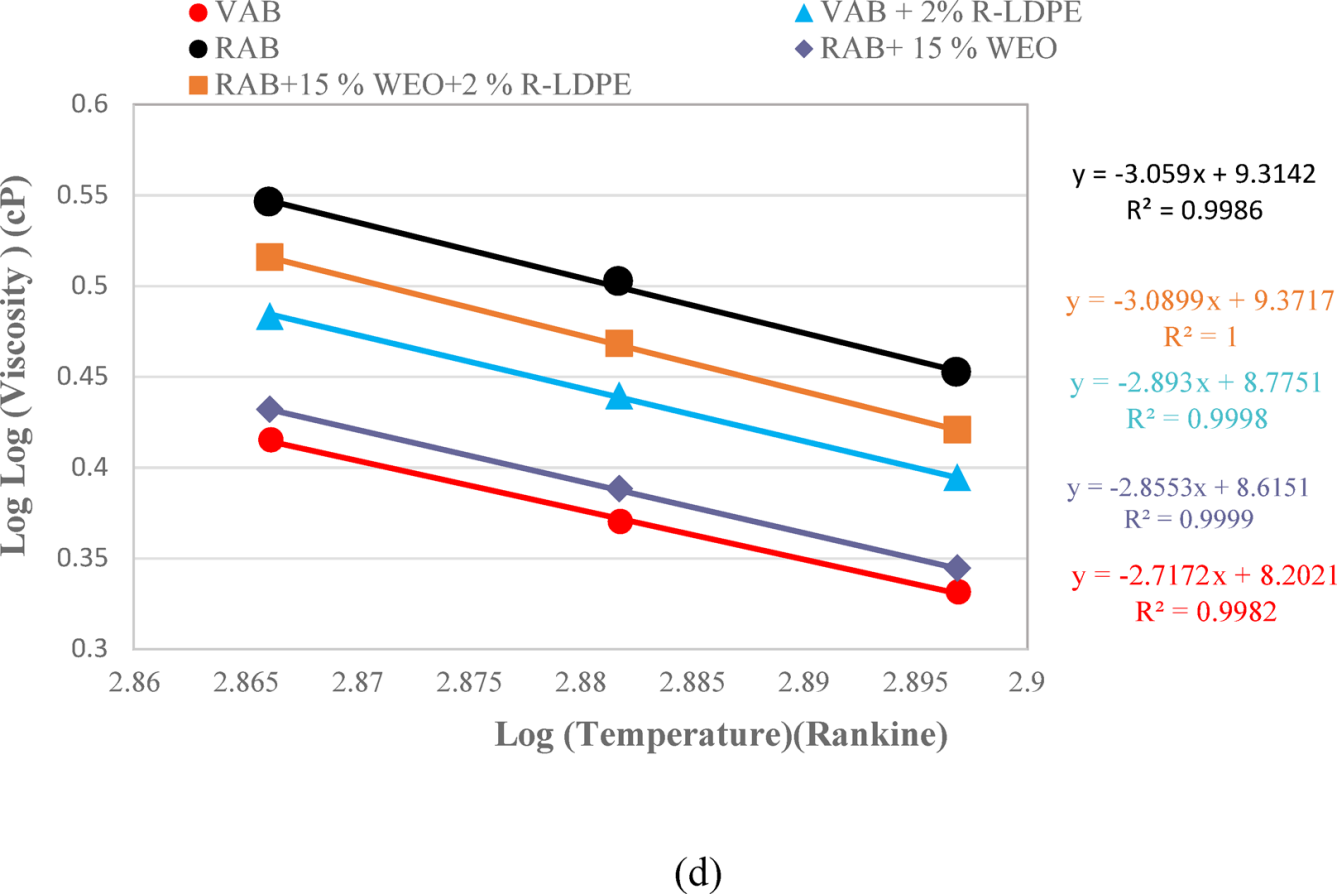
RV results and viscosity-temperature correlations of the investigated asphalt binders with optimum dosages of modifiers and rejuvenators.

The RV results, presented in Fig. 9, illustrate how the addition of R-LDPE to VAB progressively increases the viscosity of the binder as the R-LDPE content rises. This is due to a reduction in light compounds and the formation of a polymeric network within the binder, which strengthens the bonds between molecules and alters the structure of the asphalt binders towards a gel-like composition. This structural change is accompanied by an increase in asphaltene content and a decrease in aromatics, correlating with lower penetration values and higher softening points^7,65,80^.

The viscosity results revealed a highly significant effect of the modification, as indicated by an F _*critical*_ -*value* of 147 and a corresponding P-*value* of 0.0003, which is well below the significance level of 0.05. These results confirm the presence of a substantial modification effect on the asphalt binder. Specifically, the addition of 2% R-LDPE to VAB significantly enhances binder performance, as evidenced by statistically significant differences between the VAB and VAB + 2% R-LDPE groups.

In contrast, the rejuvenation of RAB with WEO leads to a noticeable decrease in viscosity, with RAB + 15% WEO showing viscosity levels close to those of VAB. However, the addition of 2% R-LDPE to 15% WEO-rejuvenated RAB results in a viscosity higher than that of both VAB and VAB + 2% R-LDPE, as depicted in Fig. 9b,c. This increase in viscosity indicates that the polymer network formed by R-LDPE has a significant impact on the modified binder’s rheological properties.

All the investigated asphalt binders satisfy the Superpave performance criteria, maintaining viscosity below 3000 cP at 135 °C, as required by ASTM D4402/D4402M-23^66^.

The incorporation of 15% EO and 2% LDBE to RAP resulted in a significant improvement in viscosity. One-way ANOVA analysis confirmed the statistical significance of this enhancement, with the F _*critical*_ -*value* of 543.56 notably exceeding the statistical F-*value* of 7.7086. Moreover, the associated P-*value* of 0.0002 validates the statistical significance of these enhancements.

#### Dynamic shear at high and intermediate temperatures

The dynamic shear testing focused on measuring two key parameters: the (G*) and (δ), which are critical for evaluating the viscoelastic behavior and stiffness of asphalt binders. The G* represents the total resistance to deformation, while δ indicates the relative proportion of elastic and viscous behavior. Using the DSR device (Anton Paar Smart Pave 102e), rheological tests were conducted on original, RTFO-aged, and PAV-aged binders to assess the rutting and fatigue performance according to ASTM D7175-23^74^. The rutting parameter, (G*/sin δ), was evaluated at high temperatures, while the fatigue parameter, (G*×sin δ), was assessed at intermediate temperatures, as per ASTM D6373-23^81^.

For high-temperature performance, the Superpave specification requires a value of (G*/sin δ) ≥ 1 kPa for the original binder and ≥ 2.2 kPa after RTFO aging. These values ensure the asphalt binders exhibit adequate resistance to deformation and viscoelastic performance under pavement conditions at high temperatures.

##### Rutting parameter (G*/sin δ)

**Fig. 10:**
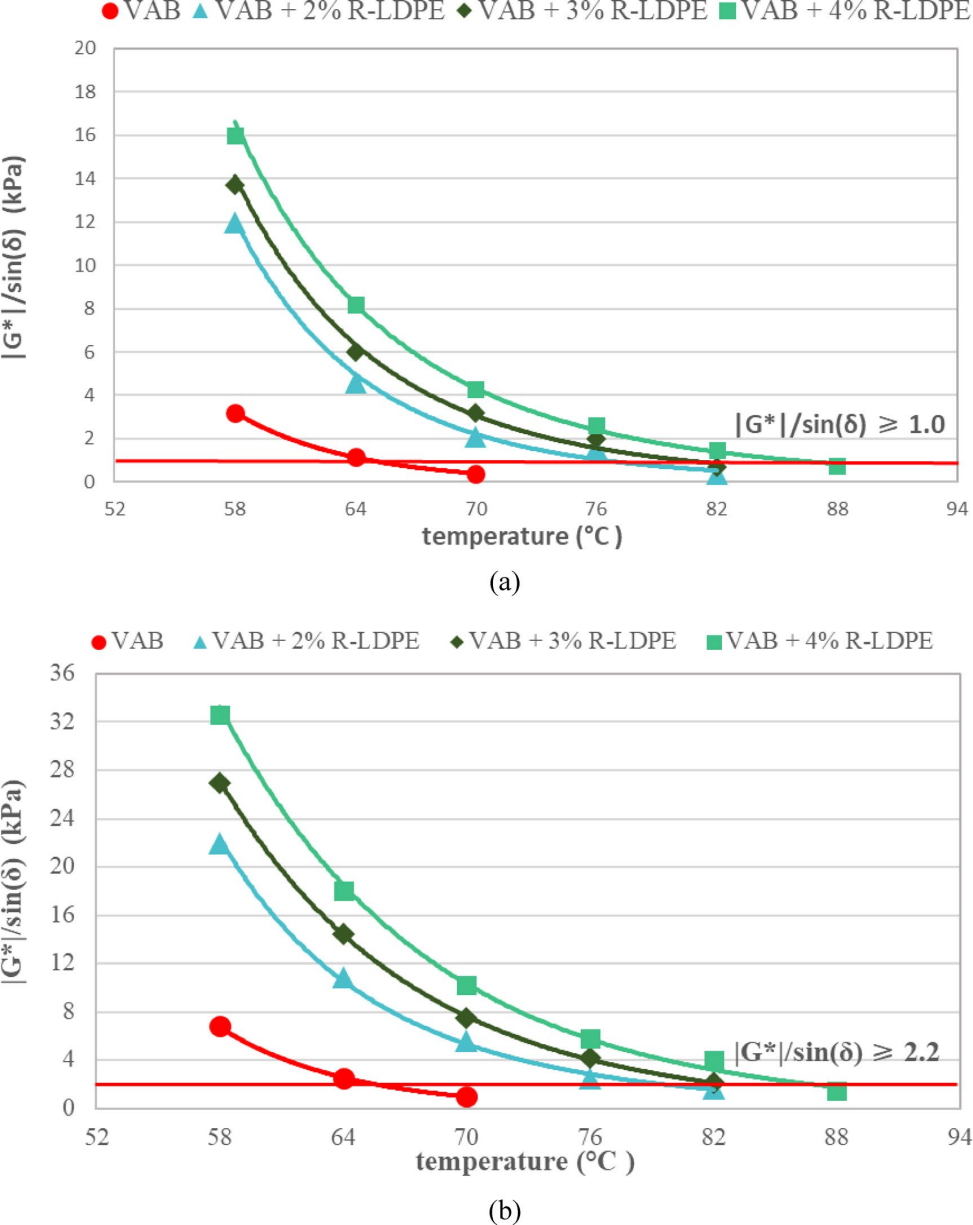

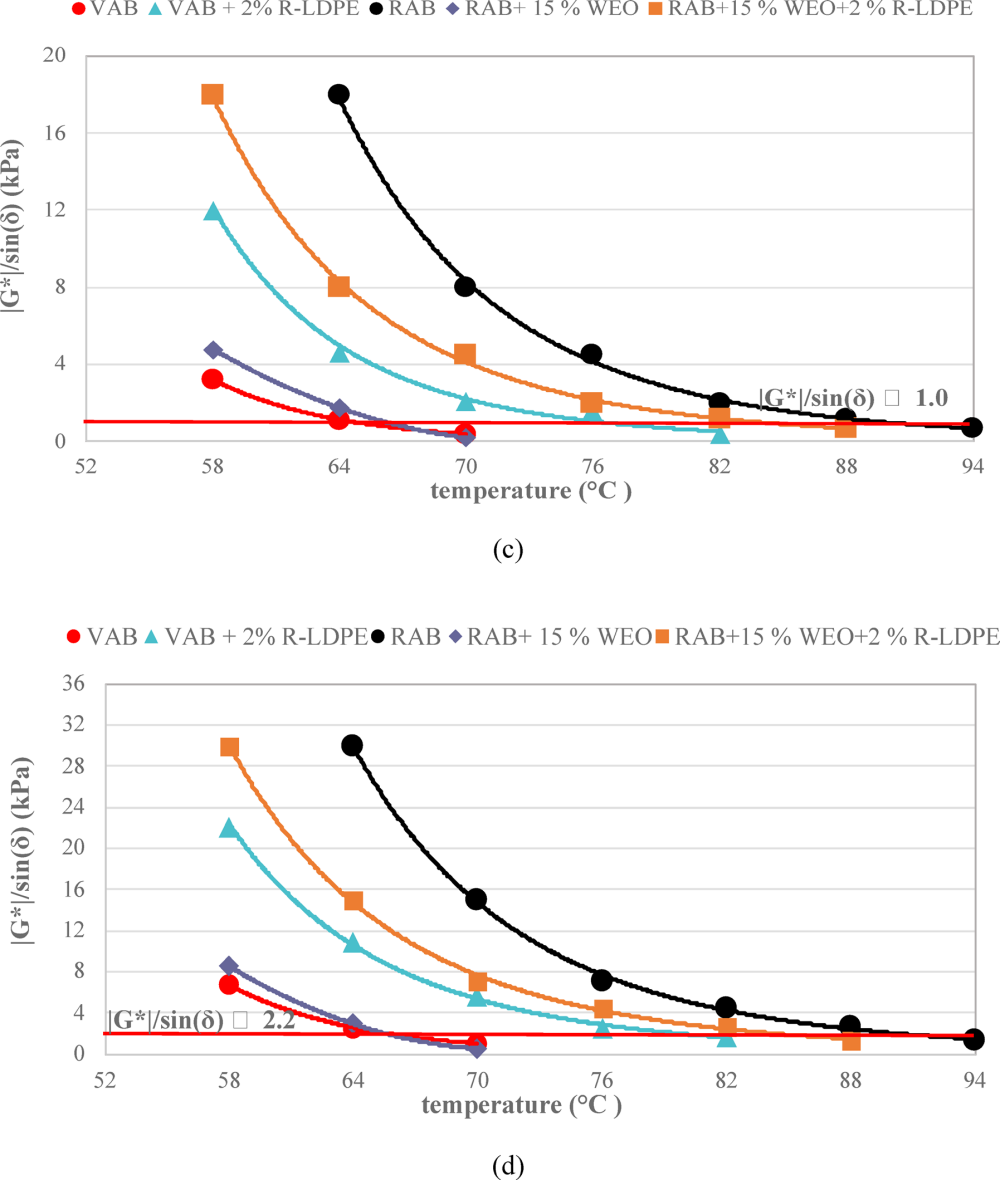
Rutting parameter results of the investigated asphalt binders with optimum dosages of modifiers and rejuvenators.

As illustrated in Fig. 10, The rutting parameter results showed that incorporating R-LDPE into asphalt binders significantly enhanced their resistance to rutting, especially under high-temperature conditions. As illustrated in Fig. 10a,b, the values of (G*/sin δ) increased for both original and RTFO-aged VAB modified with R-LDPE, indicating better elastic behaviour and reduced deformation. This improvement resulted from the development of a polymeric network within the modified binders^19,26,63–65,67,79^, providing more stiffness and elasticity^26,64^.

In particular, VAB was blended with 2% and 3% R-LDPE to achieve PG 76, ensuring its compliance for hot climates in southern Egypt^7^. Figure 10c,d illustrated the superior rutting resistance of RAB along with the associated performance grade of PG 88 after significant aging (approximately ten years). While the optimal 15% WEO content for rejuvenating RAB decreased its performance grade to PG 64, aligning closely with VAB’s required values under both original and RTFO-aged conditions, as evidenced in Fig. 10c,d. Moreover, adding 2% R-LDPE to 15% WEO-rejuvenated RAB increased its performance grade to PG 82, surpassing that of VAB + 2% R-LDPE, as shown in Fig. 10c,d. This further demonstrates the effectiveness of R-LDPE in enhancing the high-temperature performance of asphalt binders, making them more resistant to rutting and deformation.

##### Fatigue parameter (G*×sin δ)

**Fig. 11:**
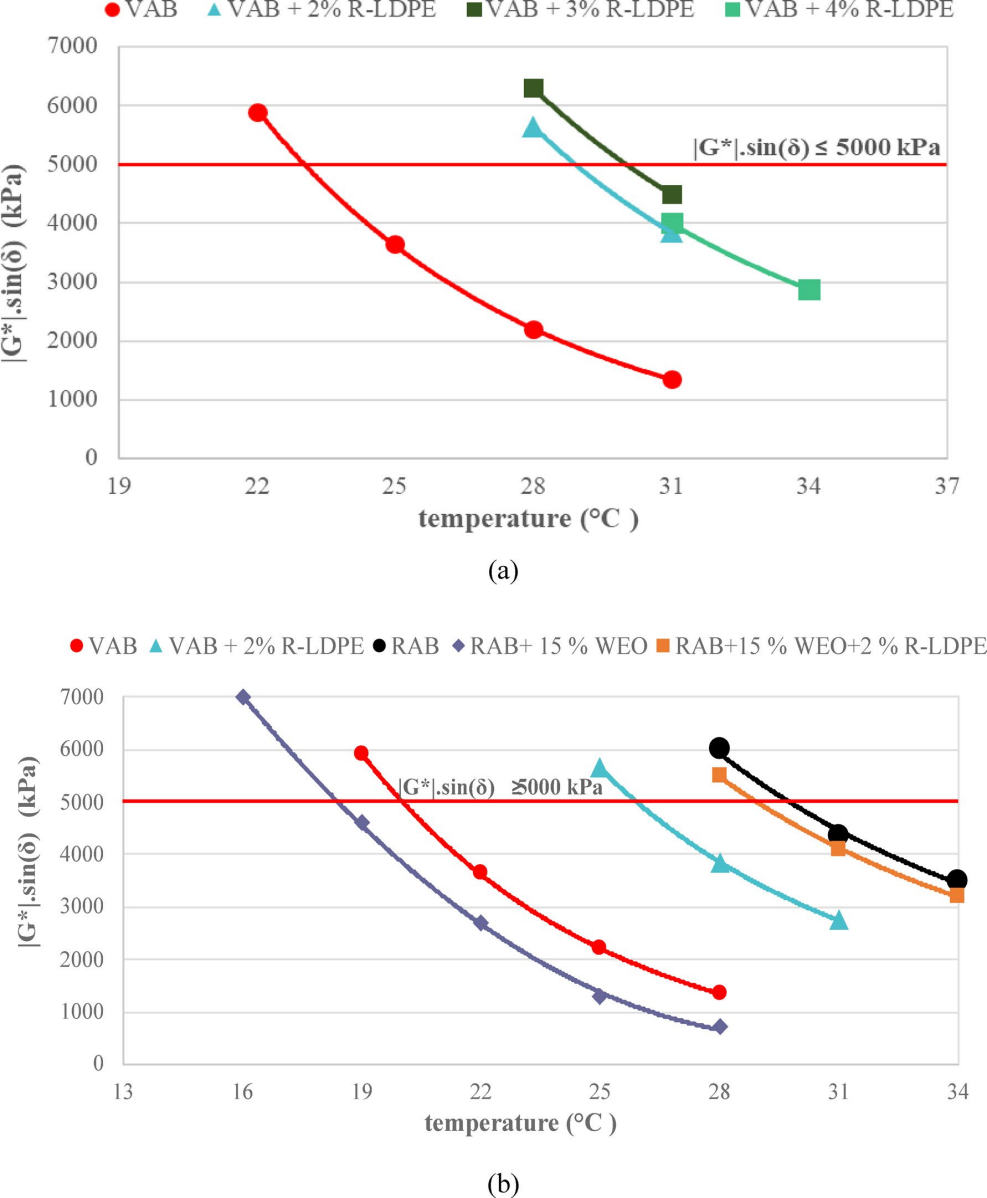
Fatigue parameter results of the investigated asphalt binders with optimum dosages of modifiers and rejuvenators.

The G*×sin δ, which measures the fatigue resistance of asphalt binders at intermediate temperatures, was assessed according to the Superpave ^TM^ asphalt binder specifications^74,81^. For long-aged binders, (G*×sin δ) should be ≤ 5000 kPa at 10 rad/s (1.59 Hz). As presented in Fig. 11a,b, the fatigue resistance of VAB and R-LDPE-modified asphalt binders after PAV aging indicated that increasing the R-LDPE content led to a reduction in fatigue resistance.

Notably, the asphalt binder modified with 2% R-LDPE exhibited a significant improvement in fatigue life, with lower (G*×sin δ) values compared to other R-LDPE-modified binders. This suggests that 2% R-LDPE offers an optimal balance between enhancing binder performance and maintaining adequate fatigue resistance.

Fatigue resistance, evaluated using the Superpave™ parameter (G*×sin δ), reveals a binder’s cracking resistance under repeated loading at intermediate temperatures^74,81^. According to Superpave™ criteria, PAV-aged binders must meet the requirement of G*×sin δ ≤ 5000 kPa at 10 rad/s (1.59 Hz). As illustrated in Fig. 11a,b, G*×sin δ values for VAB and R-LDPE-modified binders varied with polymer content. Increasing the R-LDPE dosage beyond 2% led to a rise in G*×sin δ, indicating a reduction in fatigue resistance, likely due to increased stiffness and reduced energy dissipation capacity under cyclic loading.

The binder modified with 2% R-LDPE recorded the lowest G*×sin δ among all modified samples and remained well below the 5000 kPa threshold, confirming improved fatigue resistance. This suggests that 2% R-LDPE offers an optimal balance between stiffness and flexibility, thereby enhancing the long-term performance of the binder without compromising its crack resistance.

#### Multiple stress creep and recovery (MSCR)

The MSCR test was conducted to assess the elastic and non-elastic behavior of VAB and R-LDPE-modified asphalt binders under varying stress levels. This test, following ASTM D8239-23^76^, measures two key parameters: *%R* and *J*_*nr*_. These parameters indicate the rutting resistance of RTFO-aged asphalt binders. Testing was performed at stress levels of 0.1 and 3.2 kPa, with 10 cycles at each stress level, consisting of a 1-second creep load followed by a 9-second recovery phase.

As shown in Table 3, the MSCR results demonstrate that the addition of R-LDPE to both VAB and 15% WEO -rejuvenated RAB led to a significant increase in *%R* and a decrease in *J*_*nr*_ at both stress levels. This trend highlights the enhanced rutting resistance provided by R-LDPE, as the modified binders showed stiffer behavior compared to the unmodified VAB. The addition of R-LDPE improved the elasticity and reduced the non-recoverable deformation, which is critical for better performance in high-temperature conditions and heavy traffic loads.

Statistical analysis using the one-way ANOVA test confirmed significant variation in MSCR parameters among the binder groups, demonstrating that R-LDBE and WEO contribute effectively to reducing permanent deformation and enhancing stress recovery characteristics of asphalt.

**Table 3:** MSCR results of the investigated asphalt binders with optimum dosages of modifiers and rejuvenators.

Variables		VAB	VAB + 2% *R*-LDPE	RAB	RAB + 15% WEO	RAB + 15% WEO + 2% *R*-LDPE		ASTM D8239-23^76^			
Pass/Fail Temp. (◦C)		64.8	78.9	92.7	67.9	84.9					
PG Temp. (◦C)		64	76	88	64	82					
*R*_0.1_ (%)		3.52	11.53	21.35	10.52	19.24					
*R*_3.2_ (%)		0.05	3.05	3.64	1.03	3.35					
*Jnr*_0.1_ (kPa^− 1^)		2.58	2.20	1.34	2.48	1.5					
*Jnr*_3.2_ (kPa-^1^)		2.93	1.6	2.12	2.79	1.95		**≤ 4.5**	**≤ 2**	**≤ 1**	**≤ 0.5**
*Jnr*_diff_ (%)		10.81	28.3	30.25	12.32	33.52		**Max. 75%**			
Traffic *		S	H	S	S	H		**S**	**H**	**V**	**E**

* S: stander traffic levels, H: high traffic levels, V: very high traffic levels, E: extremely high traffic levels.

#### Linear amplitude sweep (LAS)

The LAS test was conducted to assess the fatigue resistance of asphalt binders at intermediate temperatures. This test applied cyclic loading to asphalt binder specimens at a constant frequency and temperature using a DSR, following AASHTO T391 − 20^78^. Asphalt binder samples were aged using the RTFO and PAV procedures. The LAS test results were analyzed using S-VECD theory, providing insights into the durability and crack resistance of the modified binders to determine the *α* through a frequency sweep (0.20–30 Hz) and *N*_*f*_ through a linear amplitude sweep (0.1%–30% shear loading at 10 Hz for 30 min).

Table 4 provides the *N*_*f*_ and *α* values for VAB, VAB + 2% R-LDPE, RAB + 15% WEO, and RAB + 15% WEO + 2% R-LDPE at 2.5% and 5% strain. At 2.5% strain, VAB modified with 2% R-LDPE increased *Nf* by approximately 166.4%, from 9.62 million to 25.63 million cycles. Similarly, incorporating 2% R-LDPE into RAB + 15% WEO enhanced fatigue life by about 243.4%, reaching 29.36 million cycles from 8.55 million. Conversely, at 5.0% strain, modifying VAB with 2% R-LDPE decreased fatigue life by 67.4%, from 250,122 to 418,903 cycles. However, the addition of 2% R-LDPE to RAB + 15% WEO at 5% strain significantly improved fatigue life by approximately 401.4%, increasing it from 122,750 to 615,530 cycles. The intermediate temperatures for RAB + 15% WEO, VAB, VAB + 2% R-LDPE, and RAB + 15% WEO + 2% R-LDPE were 19 °C, 22 °C, 28 °C, and 31 °C, respectively. VAB modified with 2% R-LDPE achieved a performance grade of PG 76, while R-LDPE-modified WEO-rejuvenated RAB achieved PG 82. The results demonstrate that modification with R-LDPE proportion led to significant enhancements in binder’s fatigue lives^79–81^ (higher *N*_*f*_ values) at intermediate temperatures, particularly at lower strain levels (2.5% and 5.0%). This indicates that R-LDPE enhances the overall durability and binder’s resistance to cracking under specific conditions. LAS test outcomes revealed significant improvements in fatigue life, indicating greater durability and cracking resistance in binders containing R-LDBE and WEO.

**Table 4:** LAS results of the investigated asphalt binders with optimum dosages of modifiers and rejuvenators.

Variables *	VAB	VAB + 2% *R*-LDPE	RAB	RAB + 15% WEO	RAB + 15% WEO + 2% *R*-LDPE
Temp. (◦C)	22	28	31	19	31
*α*	2.614	2.752	3.250	2.586	3.168
A x 10^9^	1.271	0.955	1.496	3.071	2.95
B	-5.292	-5.852	-6.392	-6.025	-6.210
*N*_*f*_ *(2.5)*	9,621,575	25,635,481	42,785,099	8,550,575	29,362,457
*N*_*f*_ *(5.0)*	250,122	418,903	509,442	122,750	615,530

* A and B are regression parameters influenced by the characteristics of asphalt binders

In summary the interaction between RAB, WEO, and R-LDPE involves both physical and chemical aspects. The findings through morphological, thermal, chemical, rheological, and mechanical characterization confirmed this observation. Specifically, SEM and EDX analyses confirmed physical dispersion and surface interactions, while FTIR and TGA revealed chemical interactions, such as the formation of functional groups and improved thermal stability. Rheological and mechanical tests reflected the integrated effects of these interactions on binder performance.

### Statistical significance analysis

To evaluate the impact of R-LDPE and WEO additives on binder performance, a one-way analysis of variance (ANOVA) was conducted for all key performance indicators, including penetration, softening point, viscosity, MSCR parameters, and LAS-derived fatigue life values. Table 5 presents comparative results and associated statistical metrics. The ANOVA results revealed that, for each test, the calculated F-values were lower than the critical F-values, while the P-values were all ≤ 0.05. This indicates statistically significant differences among the mean values of the tested binder groups and confirms that the observed variations are not due to random chance.

The analysis provides strong evidence to reject the null hypothesis (H₀) in favor of the alternative hypothesis (H₁), affirming that the inclusion of R-LDPE and WEO led to meaningful improvements in binder performance. The consistent statistical significance across all performance categories further reinforces the effectiveness and reliability of the proposed modifications. These findings substantiate the technical feasibility of incorporating R-LDPE and WEO in asphalt binder formulations for improved rheological and durability characteristics, supporting their implementation in practical pavement applications.

**Table 5:** Comparative statistical evaluation of performance indicators.

Test		Groups	F-value	*P*-value	F_crit_-value	Effect
Penetration		VAB	7.71	0.0003	138.46	Significant differences among groups, with improvements (F ≤ F_crit_, *P* < 0.05) Alternative Hypothesis (H₁)
		VAB + 2% R-LDBE				
		RAB		0.0005	108.38	
		RAB + 15% WEO + 2% R-LDBE				
Softening		VAB		0.0005	104.89	
		VAB + 2% R-LDBE				
		RAB		0.0002	119.00	
		RAB + 15% WEO + 2% R-LDBE				
Viscosity at 135 °C		VAB		0.0003	147.00	
		VAB + 2% R-LDBE				
		RAB		0.0002	543.56	
		RAB + 15% WEO + 2% R-LDBE				
MSCR	*Jnr*_3.2_	VAB		0.0008	842.00	
		VAB + 2% R-LDBE				
		RAB		0.0002	197.00	
		RAB + 15% WEO + 2% R-LDBE				
	*Jnr*_diff_	VAB		0.0002	224.00	
		VAB + 2% R-LDBE				
		RAB		0.0007	25.66	
		RAB + 15% WEO + 2% R-LDBE				
LAS	*N*_*f*_ *(2.5)*	VAB		0.0002	176.00	
		VAB + 2% R-LDBE				
		RAB		0.0007	85.55	
		RAB + 15% WEO + 2% R-LDBE				
	*N*_*f*_ *(5)*	VAB		0.0006	29.78	
		VAB + 2% R-LDBE				
		RAB		0.0400	8.95	
		RAB + 15% WEO + 2% R-LDBE				

## Conclusions

In conclusion, this study explored the potential of using R-LDPE and WEO as modifiers and rejuvenators to enhance the performance of VAB and RAB. The experimental investigations were aimed at assessing the impact of R-LDPE and WEO on the morphological, chemical, thermal, rheological, and mechanical properties of asphalt binders, with the goal of improving their resistance to rutting, fatigue, and temperature-induced degradation.

The study demonstrated that incorporating R-LDPE and WEO into asphalt binders significantly improved their performance across several key parameters. Enhanced thermal stability was achieved through the inclusion of R-LDPE, reducing weight loss at high temperatures and delaying thermal degradation, as confirmed by TGA and FTIR analysis. The R-LDPE-modified binders exhibited higher stiffness, improved rutting resistance, with increased G*/sin δ values and elastic recovery, making them ideal for high-temperature regions with performance grades up to PG 82. The addition of R-LDPE consistently increased binder stiffness, as reflected by higher softening points and lower penetration values, while maintaining a balance with fatigue resistance at optimal levels. The rejuvenation of aged binders with WEO effectively reduced viscosity to levels comparable to virgin binders, and the combination of WEO and R-LDPE further improved stiffness and workability. Additionally, the LAS test confirmed increased fatigue life and resistance to crack propagation, underscoring the long-term durability and suitability of R-LDPE-modified binders for pavement applications. Confirming the previous results, the statistical metrics of one-way ANOVA indicated that modified asphalt binders exhibited statistically significant improvements in key performance characteristics. The incorporation of R-LDPE and WEO presents a viable approach for enhancing the thermal stability and durability of pavement structures.

Overall, this study offers valuable insights into sustainable, high-performance asphalt binders that enhance pavement durability and lifespan for both virgin and reclaimed use. The findings reveal WP modifiers, especially R-LDPE, can improve asphalt sustainability and performance while addressing waste management issues, guiding future integration of WP in road construction to promote longevity and reduce material usage.

## Future research aspects

Building on the findings of this research, several areas warrant further investigation to optimize the performance of R-LDPE-modified asphalt binders. Future studies could explore the long-term aging effects of these modified binders through extended field trials, simulating a broader range of environmental and loading conditions. Additionally, the impact of varying proportions of WEO in rejuvenated binders, alongside R-LDPE, should be examined to determine the most efficient combination for maximizing durability and cost-effectiveness.

Further rheological studies, particularly on low-temperature cracking performance, could provide insight into improving fatigue resistance without compromising flexibility. Advanced analytical techniques such as atomic force microscopy (AFM) can also be employed to better understand the microstructural changes induced by R-LDPE in the asphalt binder matrix. Lastly, the environmental and economic benefits of large-scale implementation of R-LDPE and WEO-modified binders in pavement construction should be assessed to encourage sustainable and practical applications.

## Data Availability

All data, models, and code generated or used during the study appear in the submitted article.
